# Alopecia in Harlequin mutant mice is associated with reduced AIF protein levels and expression of retroviral elements

**DOI:** 10.1007/s00335-020-09854-0

**Published:** 2020-12-26

**Authors:** Maik Hintze, Sebastian Griesing, Marion Michels, Birgit Blanck, Lena Wischhof, Dieter Hartmann, Daniele Bano, Thomas Franz

**Affiliations:** 1 Institute of Anatomy, Neuroanatomy, Medical Faculty, UKB, University of Bonn, Bonn, Germany; 2 Present Address: Medical Department, MSH Medical School Hamburg, Hamburg, Germany; 3 Dept. of Oncology, National Taiwan University Hospital, Taipei City, 100 Taiwan, ROC; 4 German Center for Neurodegenerative Diseases (DZNE), Bonn, Germany

## Abstract

We investigated the contribution of apoptosis-inducing factor (AIF), a key regulator of mitochondrial biogenesis, in supporting hair growth. We report that pelage abnormalities developed during hair follicle (HF) morphogenesis in Harlequin (*Hq*) mutant mice. Fragility of the hair cortex was associated with decreased expression of genes encoding structural hair proteins, though key transcriptional regulators of HF development were expressed at normal levels. Notably, *Aifm1 (R200 del)* knockin males and *Aifm1*^*(R200 del)/Hq*^ females showed minor hair defects, despite substantially reduced AIF levels. Furthermore, we cloned the integrated ecotropic provirus of the *Aifm1*^*Hq*^ allele. We found that its overexpression in wild-type keratinocyte cell lines led to down-regulation of HF-specific *Krt84* and *Krtap3*-*3* genes without altering *Aifm1* or epidermal *Krt5* expression. Together, our findings imply that pelage paucity in *Hq* mutant mice is mechanistically linked to severe AIF deficiency and is associated with the expression of retroviral elements that might potentially influence the transcriptional regulation of structural hair proteins.

**Supplementary Information:**

The online version of this article (10.1007/s00335-020-09854-0) contains supplementary material, which is available to authorized users.

## Introduction

As an appendage of the skin, hair follicles (HF) represent a complex miniorgan that consists of several concentric layers of epithelial cells forming the outer root sheath (ORS) in continuity with the epidermis, the inner root sheath (IRS), and the hair shaft. The latter comprises the cuticle surrounding the cortex and a central medulla. Throughout the life of an organism, HFs constantly undergo phases of growth (anagen), regression (catagen), and rest (telogen) (Schneider et al. 2009; Fuchs 2018). Several transcription factors (Jave-Suarez et al. 2002; Rogers 2004; Cai et al. 2009) control the layer-specific expression of keratins and keratin-associated proteins, which provide rigidity to the terminally differentiated cells (Langbein et al. 2004; Langbein and Schweizer 2005). The biochemical and genetic complexity of hair follicle morphogenesis is reflected by the vast number of mutant mouse lines in which pelage development is affected by mutations of various genes (Sundberg 1994; Nakamura et al. 2013). Among these determinants, recent observations indicate that aberrant mitochondrial function undermines HF development, leading to fur abnormalities (Kloepper et al. 2015; Singh et al. 2018; Stout and Birch-Machin 2019).

In this regard, the spontaneously arisen Harlequin (*Hq*) mutation was first described as an X-linked mutation causing complete alopecia in hemizygous males (Barber 1971), which was subsequently found to be associated with an endogenous ecotropic retrovirus (ERV) integration into the *Aifm1* gene (Klein et al. 2002). Preliminary observations on the histological defects of the pelage in *Aifm1*^*Hq/Y*^ hemizygous males revealed a fragility of hair shafts (Sundberg 1994).

Apoptosis-inducing factor (AIF) is a NADH- and FAD-containing oxidoreductase primarily anchored to the inner mitochondrial membrane (IMM) and facing the intermembrane space (IMS) (Susin et al. 1999). AIF has been widely described for its contribution to various cell death programs (Sevrioukova 2011; Fatokun et al. 2014; Bano and Prehn 2018). As a pro-death protein released upon mitochondrial membrane depolarization, proteolytically truncated AIF translocates to the nucleus, where it binds specific endonucleases that induce chromatin condensation and DNA fragmentation (Sevrioukova 2011; Fatokun et al. 2014; Bano and Prehn 2018). AIF has also homeostatic functions, since it contributes to the maintenance and biogenesis of the electron transport chain (ETC) (Sevrioukova 2011; Hangen et al. 2015; Bano and Prehn 2018). In this regard, AIF physically interacts with coiled-coil-helix-coiled-coil-helix domain-containing 4 (CHCHD4 or MIA40) and, as a docking site, participates in the oxidative folding of ETC subunits in the IMS (Hangen et al. 2015; Meyer et al. 2015; Bano and Prehn 2018). As previously described (Hangen et al. 2015; Meyer et al. 2015), AIF deficiency impairs CHCHD4/MIA40 stability and leads to the loss of ETC subunits and consequent aberrant mitochondrial bioenergetics (Vahsen et al. 2004). The biological contribution of AIF to mitochondrial homeostasis is further emphasized by studies in transgenic mice. While constitutive *Aifm1* knockout results in prenatal death at E8.5/E9.5 (Joza et al. 2001), genetic ablation of *Aifm1* restricted to skeletal and cardiac muscles leads to severe metabolic dysfunction and atrophy in these tissues (Joza et al. 2005). Furthermore, AIF loss induces neurodegeneration most notably in the cerebellum as initially described in the Harlequin (*Hq*) mutant mice. In this regard, the *Hq* mutation was originally identified in mice with evident baldness and late-onset ataxia (Klein et al. 2002). As a spontaneous X chromosome-linked mutation, the *Hq* allele is the result of an ecotropic proviral insertion in intron 1 of the *Aifm1* gene. In hemizygous *Aifm1*^*Hq/Y*^ males as well as homozygous *Aifm1*^*Hq/Hq*^ females, aberrant transcription of the *Aifm1* gene decreases AIF expression up to 80% (Klein et al. 2002; Bénit et al. 2008; Wischhof et al. 2018). In various tissues of *Hq* mutant mice, AIF deficiency leads to severe loss of CI subunits and, to a minor extent, decreased expression of CIII and CIV components. As result of aberrant mitochondrial bioenergetics, *Hq* mutant mice display cerebellar degeneration and myopathy between 3 and 6 months of age. Surprisingly, a different pathological profile is observed in a newly developed mouse model carrying a pathogenic AIF variant previously identified in children with encephalomyopathy (Ghezzi et al. 2010). Compared to *Hq* mutant mice, *Aifm1(R200 del)* knockin mice develop early-onset myopathy, however, they do not exhibit obvious neurological symptoms or hair loss, despite that across organs AIF protein levels are comparable between *Aifm1(R200 del)* KI and *Hq* mutant tissues (Wischhof et al. 2018). Thus, it remains a matter of further investigation, whether other biological factors, apart from AIF deficiency and consequently impaired mitochondrial function, may contribute to the phenotypes associated with the *Hq* mutation.

The bulk of the research involving the *Hq* mouse mutant mice has focused on AIF role in energy metabolism and cell death. The much more obvious pelage defect of the “Harlequin” mutant has been so far only described in few reports (Barber 1971; Sundberg 1994).

Here we report that the *Hq* allele is associated with the decreased transcription of genes specifically expressed in the hair cortex. The resulting decreased expression of hair cortex structural proteins caused mechanical weakness and finally breaking of the hair shafts below the epidermal surface level, leading to shedding of the hairs and hence baldness. Conversely, a reduction of AIF protein in the skin of *Aifm1 (R200 del)* KI mice did not result in detectable histological changes in the pelage. Notably, overexpression of the *Hq*-associated ERV in wild-type *Hoxc13*-expressing keratinocyte cell lines reduced the transcription of *Krt84* and *Krtap3*-*3*, both of which are normally expressed in the hair cortex. Together, our findings indicate that the mutant pelage phenotype of *Hq* mutant mice strongly correlates with the expression level of AIF protein. As a proof-of-principle evidence, we additionally report that the exogenous expression of *Hq* proviral sequences is sufficient to alter the transcriptional profile of cultured keratinocytes.

## Results

### Reduced AIF expression is linked to hair loss in Harlequin mutant mice

**Fig. 1:**
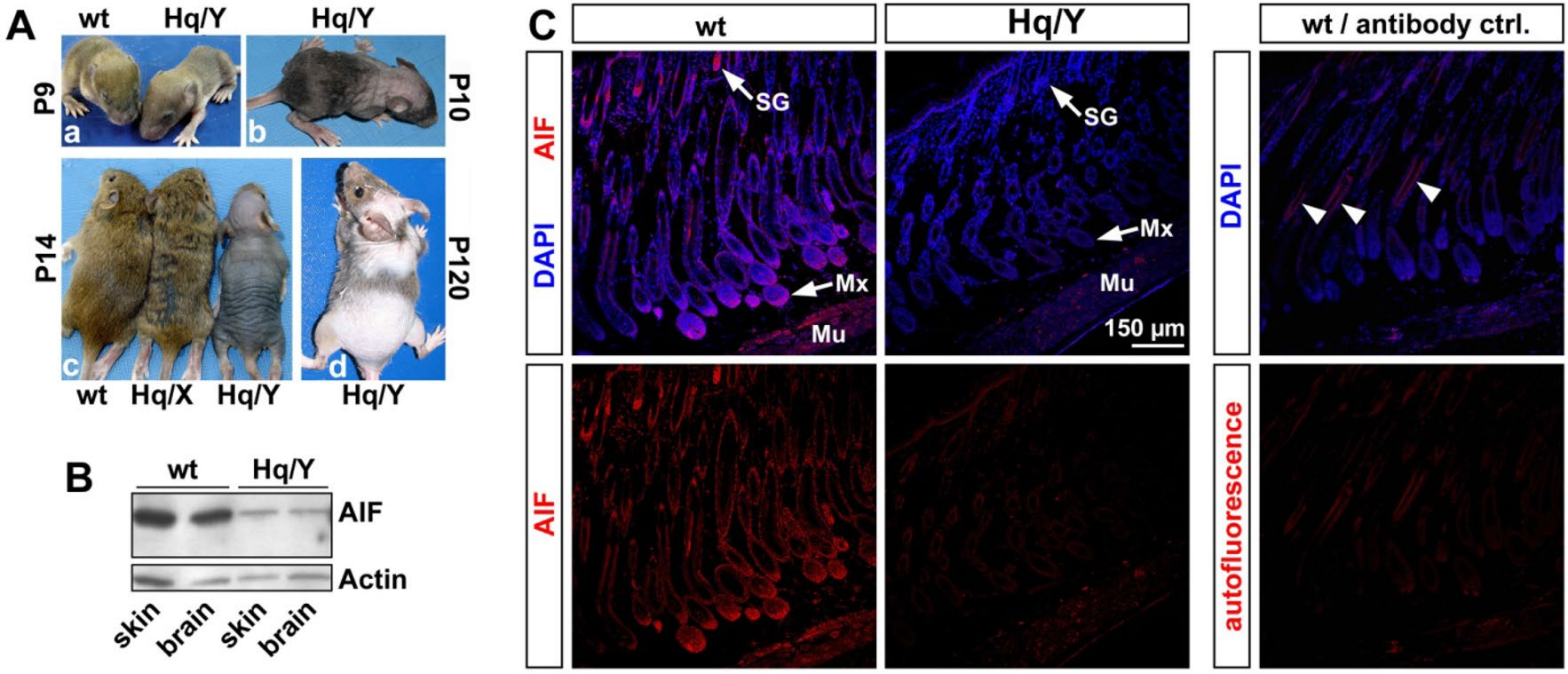
Decreased AIF level in the skin of *Hq* mutant mice is associated with hair loss. **A** Macroscopic aspect of *Hq* mutant mice showed normal hair growth until P9 (A.a). Hair loss of hemizygous *Hq* mutant males started from the head at P10 (A.b) until they appeared bald by P14, while heterozygous *Hq*/X females showed only mild pelage defects (A.c). Older *Hq* mutant males exhibited patches of pelage in various regions (A.d). **B** Western blot analysis demonstrated similar AIF reduction in brain and skin of *Hq* mutants at P12. **C** Confocal analysis of P10 skins from wt and *Hq* mutant mice. AIF immunoreactivity was most strongly detected in sebaceous glands (SG) and hair follicle matrix (Mx) as well as skeletal muscle (Mu, *Panniculus carnosus*) of wt skin, and was noticeably reduced in *Hq* mutant skin. Antibody control shows the absence of fluorescence except autofluorescence signal in the hair shafts (right panel, arrowheads)

The pelage defect of hemizygous *Aifm1*^*Hq/Y*^ mice is apparent as early as postnatal day 9 (P9), when the tips of the hair shafts became visible on the skin surface, but did not elongate further in contrast to wild-type (wt) littermates (Fig. 1A.a). During the following days, the pelage was progressively lost in the mutant across the whole body surface, beginning on the head by P10 (Fig. 1A.b). By P14, *Aifm1*^*Hq/Y*^ mice appeared bald, except for some hairs in the facial region (Fig. 1A.c). The gray color of P14 *Aifm1*^*Hq/Y*^ skin was caused by active melanogenesis, indicating that *Aifm1*^*Hq/Y*^ hair follicles (HF) remained in the anagen growth phase. Heterozygous *Aifm1*^*Hq/X*^ females (Fig. 1A.c) showed some dark stripes in their agouti coat due to the darker underfur becoming visible in areas with reduced pelage. After HF morphogenesis and the first hair cycle, *Aifm1*^*Hq/Y*^ mice developed some normal appearing, albeit shorter pelage regionally, especially in the facial region and around the root of the tail. Although variable among individual animals, pelage patches of various sizes remained on different regions of the body surface in older (P120) *Aifm1*^*Hq/Y*^ phenotypic mutants (Fig. 1A.d).

Since the *Hq* allele affects AIF expression at the mRNA level (Klein et al. 2002; Wischhof et al. 2018), we performed immunoblot analysis to assess AIF protein in *Aifm1*^*Hq/Y*^ skin. As in the brain, AIF was reduced to a similar degree in skin of *Aifm1*^*Hq/Y*^ mice compared to wt littermates (Fig. 1b). Using immunohistochemistry on P10 skin tissue sections, we observed strong AIF immunoreactivity in the hair matrix and the sebaceous glands of wt skin, which is noticeably reduced in *Aifm1*^*Hq/Y*^ skin (Fig. 1c).

### Hair follicle morphogenesis is altered in *Hq* mutant mice

**Fig. 2:**
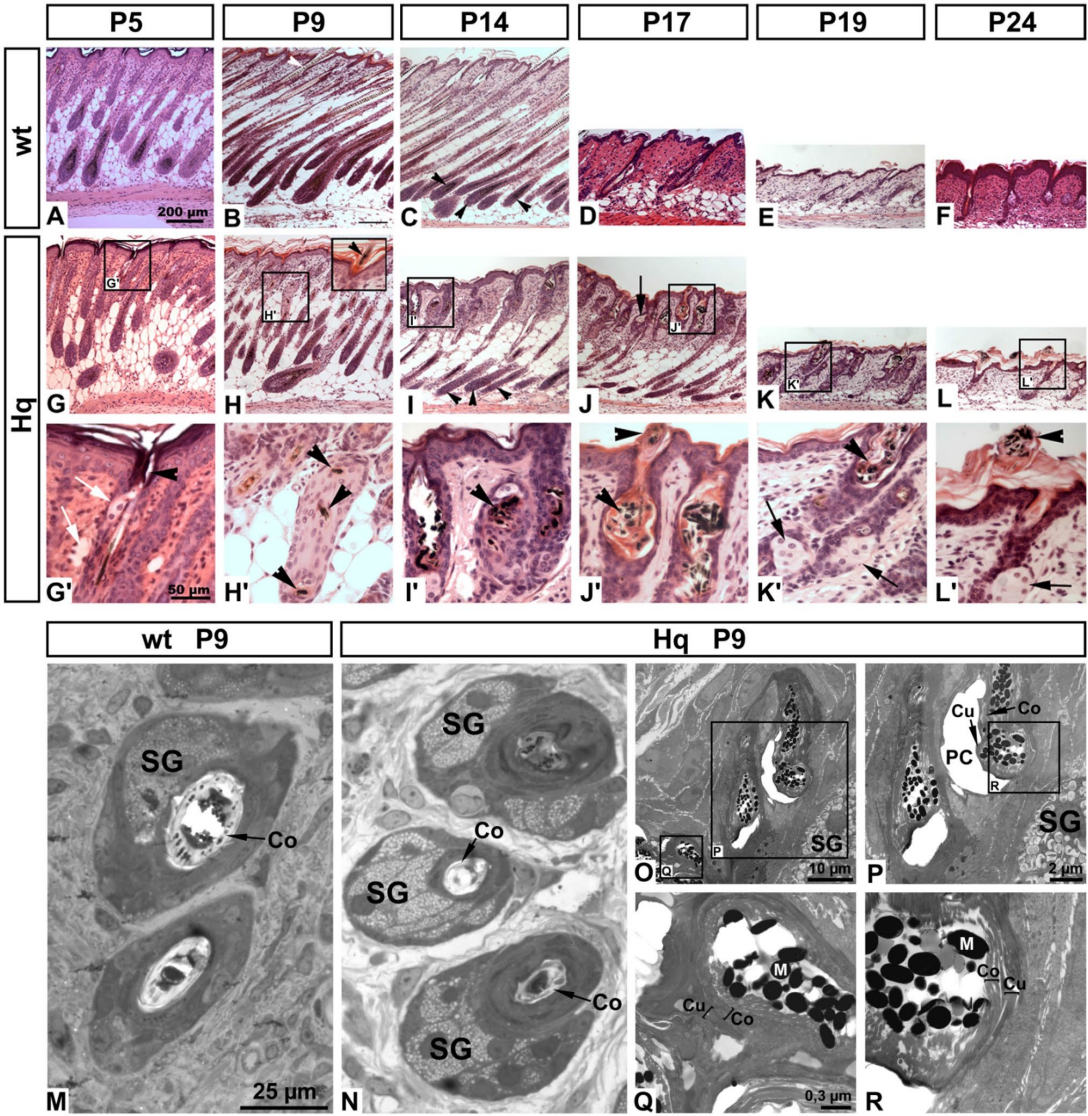
Histological analysis of *Hq* mutant hair follicle development. **a**–**l**′, H.E. stained sections of wt and *Aifm1*^*Hq/Y*^ skin revealed proper initiation of hair follicle morphogenesis in the mutant, although hair bulbs appeared slenderer in the mutant at P9 (compare arrow heads in **i** to arrow heads in **c**). Starting at P9 when wt hair shafts penetrated the epidermis (white arrow head in **b**), subapical regions of *Hq* mutant hair shafts curled up or broke subapically (arrow heads in **h**′ and **i**′), after the tip had penetrated the epidermis (arrow head in **h**). Curled up hair shafts became exteriorized between P17 and P24 (arrow heads in **j**′–**l**′). *Hq* mutant sebaceous glands appeared slightly enlarged compared to wt controls (arrows in **g**′, **j**, **k**′, **l**′). Scale bar in **a** applies also to **b**–**l**. Scale bar in **g**′ applies also to **h**′–**l**′. **m**–**r** Ultrastructural analysis of wt and mutant hair shafts. **m**, **n** Semi-thin sections showed smaller hair shaft diameter with less developed hair cortex (Co) in the mutant, while sebaceous glands (SG) were enlarged. Scale bar in **m** applies also to **n**. **o**–**r** Electron micrographs of *Hq* mutant HFs show properly developed and competent pilary canals (PC). **q**, **r** Deficient compaction of hair cortex (Co, bracket) is indicated by filamentous material, in contrast to the properly compacted hair cuticle (Cu, bracket) as indicated by homogeneous electron density. **m** Melanosomes. Scale bar in **q** applies also to **r**

We performed histological examinations of dorsal skin biopsies in *Aifm1*^*Hq/Y*^ and wt littermates. At P5, HFs were undistinguishable in number and shape between *Aifm1*^*Hq/Y*^ HF (*Hq*) and wt littermates (Fig. 2a, g). At this stage, the tips of the *Hq* hair shafts reached the preformed pilary canals (Fig. 2g′). Around P9, the earliest detectable histological difference between mutant and wt hair follicles were subapically bended hair shafts in the mutant (Fig. 2h, h′), even though the tips of mutant hair shafts could pierce through the hair canals (insert in Fig. 2h, inset). Such bending of hair shafts never occurred in age-matched wt specimens (Fig. 2b). By P14, the curled-up hair shaft material caused a widening of pilary canals in the mutants (Fig. 2i, i′). Moreover, *Hq* HFs were slenderer and showed less active melanogenesis compared to wt HFs (Fig. 2c), indicating the beginning of HF regression in the mutants. By P17, when wt HFs reached catagen with a concomitant reduction in length (Fig. 2d), *Hq* HFs still remained long with enlarged sebaceous glands (Fig. 2j). Although the openings of the pilary canals were competent, the *Hq* mutant hair shafts continued to curl up inside the pilary canals (Fig. 2j′). Thinned fragile hair shafts and enlarged sebaceous glands (Meibom gland) were also observed in the cilia of the eyelids of *Hq* mutant mice (not shown). On P19, when wt HFs had entered telogen (Fig. 2e), the *Hq* HFs still showed some epithelial trailing strand (Fig. 2k) and residues of the hair shafts in the pilary canals (Fig. 2k′). On P24, both wt and the *Hq* mutant HFs initiated the first cyclic anagen (Fig. 2f, l). Remaining hair shaft material of *Hq* mutant HFs was finally exteriorized onto the skin surface (Fig. 2l′).

Sections of P9 skin showed thinner hair shaft diameter and enlarged sebaceous glands in *Hq* mutants (Fig. 2n) compared to the wt (Fig. 2m). Electron microscopic examinations of *Hq* HFs revealed that the pilary canal was open and cuticles of hair shaft and inner root sheath were both intact (Fig. 2o, p). However, the hair cortex contained filamentous material indicating incomplete keratinization, in contrast to the adjacent hair cuticle which appeared homogeneously electron dense and thus fully keratinized (Fig. 2q, r).

Our morphological observations indicate that in *Hq* hemizygous mutant males, HFs produce hair shafts that are mechanically less rigid compared to wt controls, possibly because of keratinization defects in the subapical hair cortex.

### *Hq* mutant HFs exhibit a decreased expression of genes involved in hair cortex-specific keratinization

**Fig. 3:**
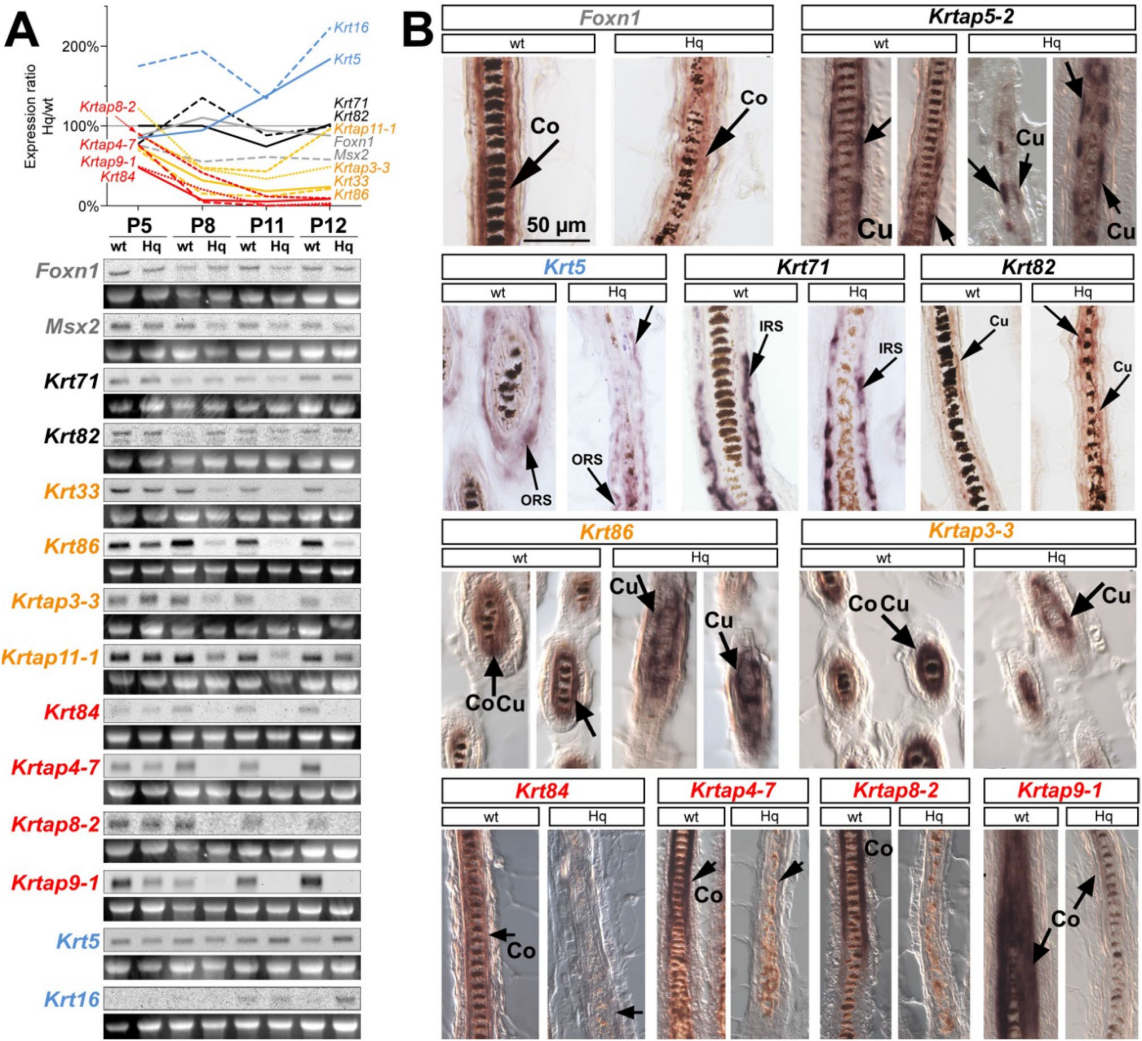
Dysregulated hair cortex-specific structural gene expression in *Hq* mutant hair follicles. **a** Northern blot analyses of genes associated with hair and skin development demonstrated stable expression of hair cortex-specific regulators *Foxn1* and *Msx2* in *Hq* mutant skin during anagen between P5-P12 (shown in gray). Structural genes fall into four categories with respect to expression level changes in *Hq* mutant skin compared to wt littermates: some were not altered (black), some were partially lost (orange), and some were completely lost in the mutant (red), while *Krt5* and *Krt16* were up-regulated in the mutant (light blue). Line graph illustrates expression levels in *Hq* mutant skin relative to age-matched wt littermate samples, Northern blot results and respective 28S rRNA bands of the gel images as loading controls are shown below. **b** In-situ hybridization revealed that expression of the hair cortex-specific regulator *Foxn1* was maintained in *Hq* mutant HFs. However, hair cortex-specific structural genes were lost in the mutant, while expression of genes in other layers of the hair follicle was not affected. Co, hair cortex; Cu, hair cuticle; IRS, inner root sheath; ORS, outer root sheath

Using Northern blot analysis, we investigated the expression of genes encoding regulatory and structural hair proteins in postnatal pups (i.e., P5-P12), within a time window in which the hair shaft fragility became histologically evident in *Hq* mutant HFs. The transcription factors *Foxn1* and *Msx2*, essential regulators of HF morphogenesis, were similarly expressed in *Hq* mutant and wt skin at P5, P8, P11, and P12 (Fig. 3a, gray labels). Because the layer-specific transcriptional regulation of structural genes within the hair follicle is not well understood (Rogers 2004), we next compared the transcription levels of multiple genes encoding hair keratins and keratin-associated proteins (*Krtaps*) between *Hq* mutant and wt skin. We observed that the analyzed genes fall into four categories, while *Krt71* and *Krt82* remained unchanged (Fig. 3a, black labels), over time other hair-specific structural genes were partially (*Krt33*, *Krt86*, *Krtap3*-*3*, *Krtap11*-*1*; Fig. 3a, orange labels) or completely lost (*Krt84*, *Krtap4*-*7*, *Krtap8*-*2*, *Krtap9*-*1*; Fig. 3a, red labels) in *Hq* mutant skin. By contrast, epidermal *Krt5* and wound healing-related *Krt16* appeared to be up-regulated in *Hq* mutant skin (Fig. 3a, light blue labels), consistent with the reactive thickening of the epidermis in *Hq* mutant mice compared to wt littermates.

To more precisely localize the dysregulated genes within the mutant HFs, we performed in situ hybridization and found that the down-regulation of hair structural genes specifically occurred in the hair cortex (Fig. 3b), consistent with the deficient hair cortex keratinization. *Krt84*, *Krtap4*-*7*, *Krtap8*-*2*, and *Krtap9*-*1* genes were detectable only in the hair cortex of wt HFs and were all completely lost in *Hq* mutant HFs. *Krt86* and *Krtap3*-*3* genes were reduced, although they were still detectable due to residual expression in the cuticle of *Hq* mutant HFs, while their cortical expression domain was lost. *Krt5*, *Krt71*, *Krt82*, and *Krtap5*-*2* genes, which are normally expressed in other layers of the HF, as well as the hair cortex-specific regulatory gene *Foxn1*, were comparably expressed in *Hq* mutant and wt HFs.

These results suggest that the *Hq* mutation causes the progressive down-regulation of distinct *Krt* and *Krtap* genes specifically in the hair cortex, although normal expression levels and localization of hair cortex-specific regulatory genes *Foxn1* and *Msx2* in *Hq* mutant skin indicate that the cells forming the hair cortex are not lost in mutant HFs.

### Reduced AIF protein expression is not associated with alopecia in *Aifm1 (R200 del)* KI mice

**Fig. 4:**
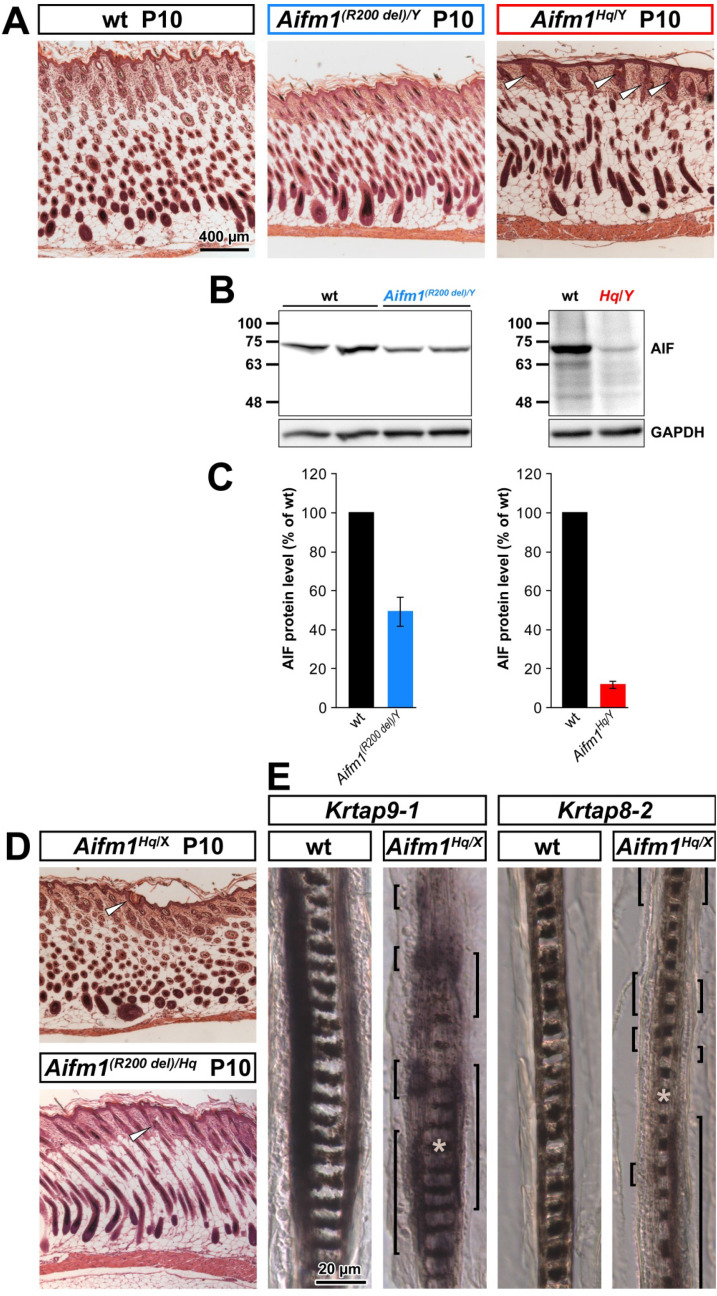
Mild hair defects in heterozygous *Aifm1*^*Hq/X*^ and *Aifm1*^*(R200 del)/Hq*^ mice. **a** H.E. stained skin sections demonstrated no broken hair shafts at P10 in *Aifm1*^*(R200 del)/Y*^ skin, while almost all hair shafts were broken in *Aifm1*^*Hq/Y*^ skin (white arrow heads). **b** Western blot showed reduced AIF protein levels in *Aifm1*^*(R200 del)/Y*^ skin, although not as dramatically as in *Aifm1*^*Hq/Y*^ skin. **c** Quantification of B. **d** Only very few broken hair shafts (white arrow heads) were found in H.E. stained skin sections in *Aifm1*^*Hq/X*^ skin at P10, which was not exacerbated in *Aifm1*^*(R200 del)/Hq*^ skin. **e** In-situ hybridizations revealed interrupted expression domains of hair cortex-specific genes *Krtap9*-*1* and *Krtap8*-*2* (expression domains indicated by brackets). White asterisks indicate melanin granules in the hair medulla, demonstrating longitudinal sections through the middle of the hair shafts

The *Aifm1*^*Hq*^ mutation is caused by the insertion of an endogenous retrovirus (ERV) genome into the *Aifm1* locus, leading to reduced *Aifm1* transcription and protein expression (Klein et al. 2002). To dissociate the in vivo consequence of AIF deficiency from the *Hq*-linked ERV, we employed *Aifm1 (R200 del)* KI mice. As previously described (Wischhof et al. 2018), these mice are a tractable model of an inherited form of human encephalomyopathy in which a single amino acid is lost in the AIF protein. We performed immunoblot analyses (Fig. 4b, c) in the skin of *Aifm1 (R200 del)* KI males compared to age-matched wt littermates. We found an AIF protein reduction (~ 49 ± 8%, *n* = 3) which was milder compared to *Aifm1*^*Hq/Y*^ males (12 ± 2% of wt levels, n = 4). More importantly, we did not notice macroscopic or histological pelage defects in *Aifm1 (R200 del)* KI males (Fig. 4a). Thus, we crossed *Aifm1 (R200 del)* KI males with *Aifm1*^*Hq/X*^ females to generate a small cohort of double-heterozygous *Aifm1*^*(R200 del)/Hq*^ females in which we histologically investigated the skin during anagen. In principle, the combination of the two alleles would lead to a more severe reduction of AIF protein levels than in *Aifm1*^*Hq/X*^ females, further approximating the uniformly low levels detected in *Aifm1*^*Hq/Y*^ hemizygous males. We hypothesized that this might lead to exacerbated alopecia in *Aifm1*^*(R200 del)/Hq*^ females compared to *Aifm1*^*Hq/X*^ heterozygous females. Interestingly, we found that double-heterozygous *Aifm1*^*(R200 del)/Hq*^ females did not exhibit more frequent or more severe hair follicle defects at P10 than heterozygous *Aifm1*^*Hq/X*^ mice (Fig. 4d).

We were very intrigued by the mild pelage phenotype in heterozygous *Hq* females. Thus, we investigated the expression of hair cortex-specific *Krtap8*-*2* and *Krtap9*-*1* genes using in situ hybridizations. Each HF is of polyclonal origin and random X-chromosome inactivation in each cell clone is inherited by its progeny (Legué and Nicolas 2005). Hence, each HF of heterozygous female *Aifm1*^*Hq/X*^ mice is predicted to contain a mosaic of cells expressing wt and mutant alleles. On P14, when all HFs of hemizygous *Aifm1*^*Hq/Y*^ males exhibited structural defects and enlarged sebaceous glands (see Fig. 2i), only few HFs of heterozygous *Aifm1*^*Hq/X*^ females showed deformed hair shafts, though many more had noticeably enlarged sebaceous glands (data not shown). In situ hybridizations revealed that the hair cortex-specific *Krtap9*-*1* and *Krtap8*-*2* genes in the *Aifm1*^*Hq/X*^ heterozygous females exhibit discontinuous expression domains (Fig. 4e), resembling clonally derived segments of the hair shaft (Sequeira and Nicolas 2012). Such pattern of structural hair cortex gene expression seems to be sufficient to provide for hair shaft rigidity in most HFs.

Taken together, reduction of AIF protein levels in *Aifm1 (R200 del)* KI mutant animals to as low as 50% of wt levels is not sufficient to impair HF development. Moreover, the mild pelage defect of heterozygous *Aifm1*^*Hq/X*^ females is explained because each HF contains a mix of cell populations in which hair gene expression was either lost or completely preserved, rather than evenly reduced hair cortex gene expression in all cells of every HF. This mixed gene expression pattern in heterozygous *Aifm1*^*Hq/X*^ HFs suggests a cell-autonomous mechanism for the impairment of hair gene expression in the mutant.

### Retroviral elements are detectable in *Hq* mutant skin

**Fig. 5:**
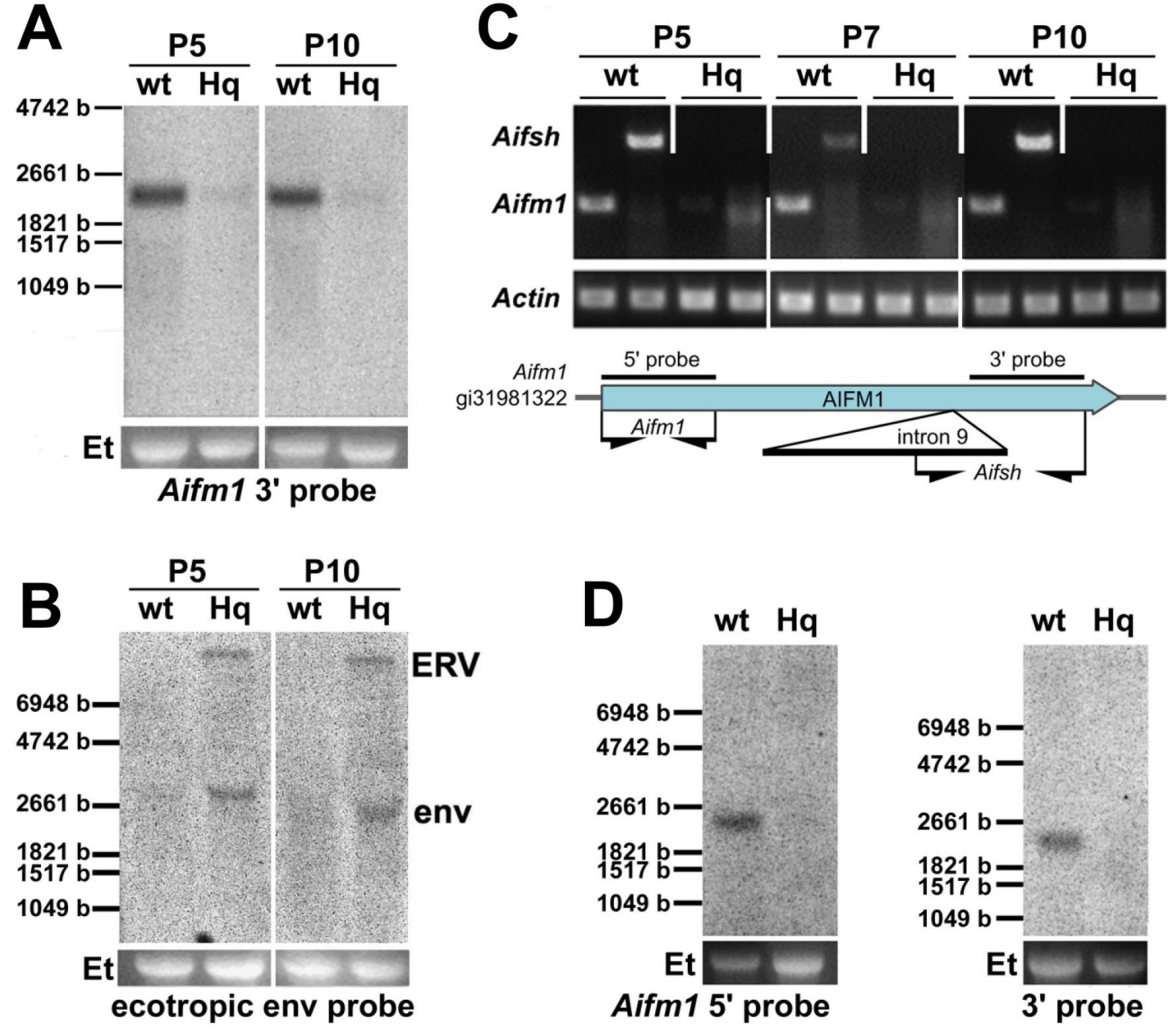
Expression of *Aifm1* and retroviral elements in *Hq* mutant skin. **a** Northern blot analysis showed reduced expression of *Aifm1* transcript in *Hq* mutant skin. **b** Retroviral elements are detectable by Northern blot analysis in *Hq* mutant skin but not in wt controls. **c** *Aifm1* full-length or *Aifsh* transcripts are detected by RT-PCR in wt skin, but not in *Hq* mutant skin. Location of PCR primers relative to *Aifm1* full-length transcript is indicated below. **d** Northern blot analysis using probes against the 5′-end (left panel) or the 3′-end (right panel) of the *Aifm1* transcript showed no detectable expression of uncharacterized transcripts in *Hq* mutant skin. Et indicates 28S rRNA bands of the respective gel images as loading controls

RNA blots of P5 and P10 wt and *Hq* mutant skin hybridized with a probe specific for the 3′ end of the *Aifm1* transcript showed a dramatic decrease of *Aifm1* transcription at P5 and P10 (i.e., before and after the observed histological changes in the mutant HF; Fig. 5a). We also analyzed the expression of known *Aifm1* splice forms. Using primers specific for the 5′ end of the *Aifm1* full-length and the *Aifsh* isoform (Delettre et al. 2006a, b), we performed RT-PCRs on skin cDNA preparations of *Hq* mutant and wt mice at P5, P7 and P10. At the three tested time points, expression of full-length *Aifm1* and *Aifsh* transcripts in *Hq* mutant skin was not detected using our PCR conditions (Fig. 5c). RNA blots from P10 *Hq* mutant and wt skin hybridized with two *Aifm1*-specific probes derived from either the 5′ or the 3′ end (as indicated in the sketch in Fig. 5c) also showed no detectable expression of any other alternative *Aifm1* transcripts in *Hq* mutant skin (Fig. 5d). Based on this line of evidence, no known or uncharacterized *Aifm1* splice variants are expressed in the skin of *Hq* mutant mutant mice.

We next hybridized the same wt and *Hq* mutant skin RNA samples using a probe specific for ecotropic *env* (envelope) sequences. We detected abundant expression of a full-length endogenous ecotropic retrovirus transcript (ERV, appr. 8 kb) and a spliced *env* transcript (approx. 2.5 kb) in the skin of *Hq* mutant mice, but not in skin RNA of wt littermates (Fig. 5b). We compared the expression of retroviral transcripts in cDNA samples of skin and cerebellum using semi-quantitative RT-PCR. We found strong expression of *env* and moderate expression of the *gag*-*pol* transcript in both *Aifm1*^*Hq/Y*^ hemizygous males and *Aifm1*^*Hq/X*^ heterozygous females, while both transcripts were barely detectable in wt littermates (Supplementary Fig. S1A). To confirm that the detected transcripts originated from the *Aifm1*^*Hq*^ allele-associated ERV, we sequenced the transcripts and found 100% identity to the *Hq*-ERV genome (Supplementary Fig. S1B) inserted into the *Aifm1*^*Hq*^ allele (see below). These results indicate that endogenous ecotropic retroviral transcripts are abundantly expressed in skin and cerebellum of hemizygous and heterozygous *Hq* mutants, but not in wt littermate controls.

### Overexpression of *Hq*-associated ERV represses *Krtap3*-*3* and *Krt84* in cultured cells

**Fig. 6:**
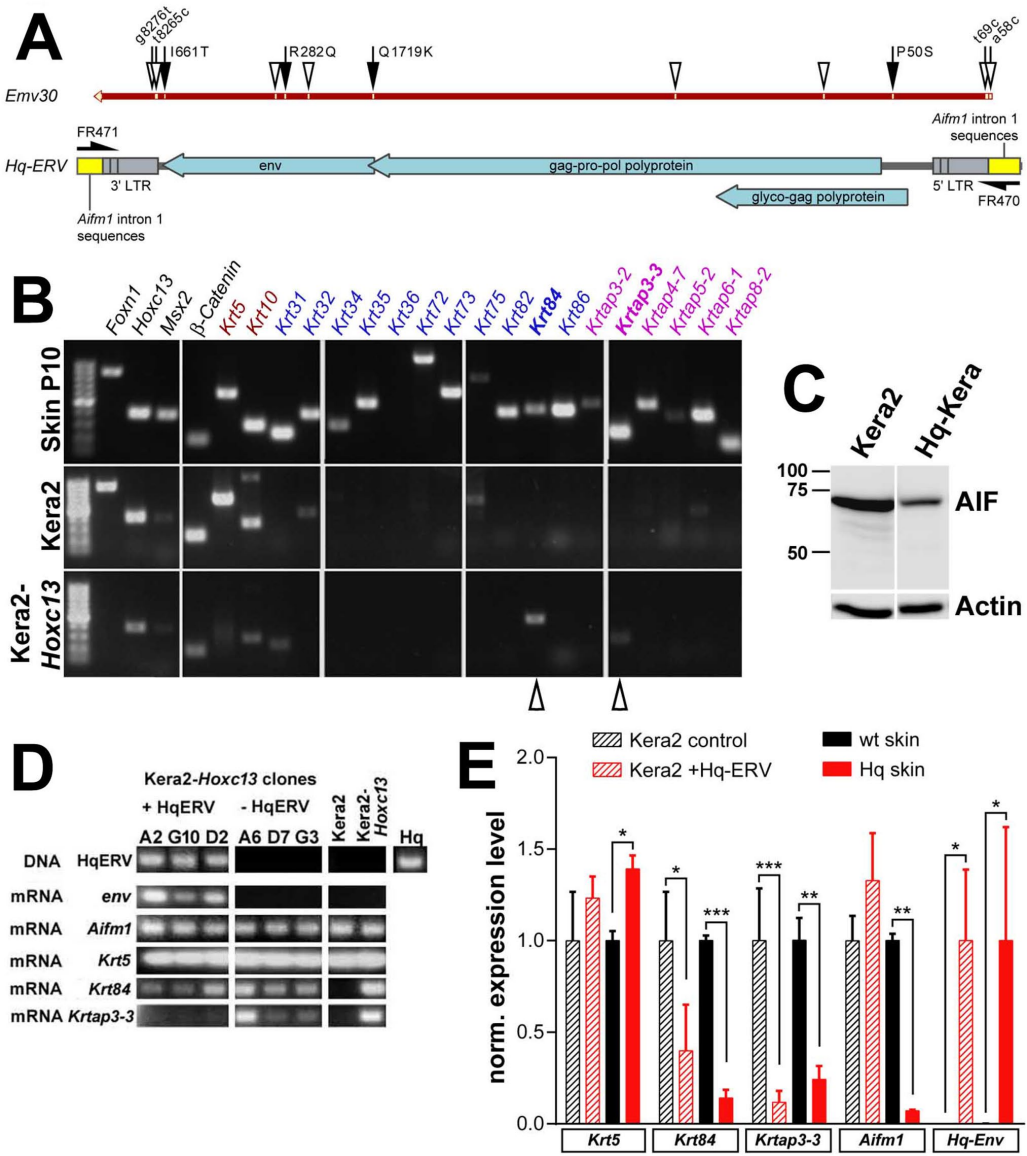
Expression of the *Hq*-linked retrovirus suppresses structural hair gene expression in cultured keratinocytes. **a** sequencing of the *Hq* allele-related retrovirus revealed a complete endogenous retroviral genome highly homologous to Emv30. Single-nucleotide changes within the *Hq*-ERV genome with respect to Emv30 are indicated (black arrows: non-conservative nucleotide changes within coding regions; white arrows: nucleotide changes in non-coding regions; white arrow heads: conservative nucleotide changes within coding regions). **b** RT-PCR analysis of HF-related genes in Kera2 and Kera2-*Hoxc13* keratinocytic cell lines. Transcript names indicated above are color-coded by category (black: regulators of HF development; red: epidermal keratins; blue: HF-specific keratins; pink: HF-specific keratin-associated protein genes). Epidermal keratins were down-regulated, while HF-specific *Krt84* and *Krtap3*-*3* were up-regulated in Kera2-*Hoxc13* cells (white arrow heads). **c** Western blot demonstrated AIF deficiency in *Hq* mutant keratinocytic cell line Hq-Kera, similar to AIF reduction in skin (see Fig. 1B). **d** Semi-quantitative RT-PCR analysis revealed reduced expression of structural hair genes *Krt84* and *Krtap3*-*3* in *Hq*-ERV-transfected Kera2-*Hoxc13* clones compared to clones lacking *Hq*-ERV, while *Aifm1* expression was not changed. **e** qRT-PCR analysis demonstrated reduction of *Krt83* and *Krtap3*-*3* in *Hq*-ERV-transfected keratinocytic cells, similar to *Hq* mutant skin. *Aifm1* was not reduced in *Hq*-ERV-transfected keratinocytic cells, in contrast to *Hq* mutant skin where *Aifm1* expression level was reduced by almost 90% compared to wt controls

To assess the putative influence of the *Hq*-associated ERV on the expression of hair cortex-specific genes, we cloned and sequenced the entire endogenous retroviral genome, including some flanking sequences derived from the first intron of the *Aifm1* locus (Supplementary sequence). The *Hq*-associated ERV sequence is highly similar to the published sequence of the *Emv30*-like endogenous retrovirus (Triviai et al. 2014), differing only in 12 nucleotides (Fig. 6a), including 2 non-synonymous variations each in the *gag*-*pol* region (*glyco*-*gag* P50S; *gag*-*pro*-*pol* Q1719K), and in the *env* region (R282Q and T661I) as well as 4 synonymous mutations (*gag*-*pol* E179, V648; *env* P207). We also identified two single-nucleotide changes within a span of 12 nucleotides at the 5′ start of the U3 regions in the viral 5′ and 3′ LTRs.

To investigate the effect of *Hq*-associated retroviral expression, we established a permanent keratinocyte cell line from C3H/FeB wt skin using a retroviral vector transducing a temperature-labile SV40 T gene (Jat et al. 1986; Jat and Sharp 1989). Transcriptional profiling of the wt keratinocyte cell line Kera2 revealed that these cells expressed most of the epidermal genes, but no hair follicle-specific genes (Fig. 6b). We therefore derived clones of Kera2 cells stably transfected with a *Hoxc13* expression vector to induce the expression of HF-specific genes (Jave-Suarez et al. 2002), knowing that the expression of hair shaft-specific genes depends on a delicate balance of *Hoxc13* expression levels (Godwin and Capecchi 1998, 1999; Tkatchenko et al. 2001). We chose one clone of Kera2-*Hoxc13*-expressing cells that showed detectable levels of hair cortex-specific *Krt84* and *Krtap3*-*3* gene expression (Fig. 6b, arrowheads), which are down-regulated in *Hq* mutant skin (see Fig. 3). Immunoblot analysis demonstrated that decreased AIF protein was persistent in a keratinocyte cell line *Hq*-Kera which was obtained from *Hq* mutant skin (Fig. 5c). Since these immortalized cells still exhibit AIF loss, we used Kera2-*Hoxc13* cells to investigate whether changes of HF-specific genes would occur in the presence of *Hq*-associated ERV sequences.

Kera2-*Hoxc13* cells were co-transfected with the entire cloned *Hq*-ERV (including some flanking sequences from the *Aifm1*^*Hq*^ genomic insertion site), together with a puromycin resistance gene for the selection of transfected cells. The transfected *Hq*-ERV genome was detected in three of the selected puromycin-resistant clones, while three other puromycin-resistant clones lacked the *Hq*-ERV proviral genome and were henceforth used as negative controls (Fig. 6d). ERV expression in all three *Hq*-ERV-positive clones was confirmed by RT-PCR using primers specific for ecotropic *env* sequences (Fig. 6d). Further expression analyses demonstrated that ecotropic *env* expression did not affect the expression of *Aifm1* and *Krt5*, which is normally expressed in the epidermis and the outer root sheath (Fig. 3). Conversely, two of three Kera2-*Hoxc13* clones carrying the *Hq*-ERV sequences showed reduced expression of hair-specific *Krt84* gene compared to the *Hq*-ERV-negative clones, and all three Kera2-*Hoxc13* clones carrying the *Hq*-ERV sequences lacked expression of *Krtap3*-*3*, which was readily detectable in the *Hq*-ERV-negative controls (Fig. 6d).

We quantified the expression levels of *Krt5*, hair cortex-specific *Krt84* and *Krtap3*-*3*, as well as *Aifm1* and ecotropic *env* sequences by qRT-PCR in Kera2-*Hoxc13* clones that did or did not carry at least one genomic copy of the *Hq*-ERV genome. We found that *Aifm1* as well as *Krt5* expression levels were not reduced in *Hq*-ERV transfected clones regardless whether they expressed *env* or not (Fig. 6e). In contrast, *Krt84* and *Krtap3*-*3* expression were reduced in *Hq*-ERV-positive, but not in the control clones, thereby reflecting their dysregulation in vivo in the skin. Based on the data obtained using these cultured cells, down-regulation of hair cortex genes *Krtap3*-*3* and *Krt84* was associated to the expression of ERV-derived sequences. Our data suggest that the proviral insertion in the *Aifm1* gene of the *Hq* mutant mice suppresses *Aifm1* expression and might interfere with transcriptional programs involved in hair shaft development, possibly contributing to *Hq*-associated alopecia.

## Discussion

The exploration of mouse mutations causing alterations of hair texture and alopecia has elucidated many molecular aspects of hair biology (Nakamura et al. 2013). This study shows that the pelage defect of the “Harlequin” (*Hq*) mutant is caused by the fragility of subapical parts of the hair shafts. We showed that the transcription of genes encoding structural proteins is specifically lost in the hair cortex of *Hq* mutant male HFs, causing the hair shafts to curl up inside the pilary canal.

The X chromosome-linked *Hq* mutant arose spontaneously in 1971 and was identified by its pelage defect (Barber 1971). The pelage defect was associated with fragility of subapical parts of the hair shafts, resulting in complete baldness of male hemizygotes (Sundberg 1994). In this respect it differs from the *nude* (*Foxn1*^*nu*^) mutants, in which the hair shafts are fragile in their entirety and never appear on the skin surface (Mecklenburg et al. 2001). Moreover, scanning electron microscopy revealed that the twisted parts of the *nude* hair shafts lacked a cuticle (Mecklenburg et al. 2001), while in *Hq* mutant males the cuticle was morphologically preserved and fully keratinized, even where it was bent in the pilary canal. Thus, *Aifm1* down-regulation by the *Hq* mutation in the hair follicle causes alopecia by increasing the fragility of the subapical hair cortex and hence is distinct from the *nude* and related phenotypes. This distinction is corroborated by the normal expression of *Foxn1* in *Aifm1*^*Hq/Y*^ mice during hair follicle morphogenesis.

The *nude* phenotype is caused by mutations of the transcription factor gene *Foxn1* (Schlake et al. 1997; Mecklenburg et al. 2001), which regulates the expression of genes encoding structural proteins in the hair shaft (Schlake et al. 2000; Mecklenburg et al. 2001; Schweizer et al. 2007). Other genes influencing the transcriptional activation by *Foxn1* such as *Msx2* and *Hoxc13* produce similar effects on hair shaft morphology and hair cycling (Ma et al. 2003; Potter et al. 2011). Here we show that, in *Hq* mutant mice, the transcription of *Foxn1* and *Msx2* is not altered during the period of hair follicle morphogenesis when hair fragility is apparent, while some of their target genes, specifically hair keratin genes and genes encoding keratin-associated proteins, are down-regulated. Thus, the reduced expression of AIF by the *Hq* mutation interferes with the expression of structural genes specifically in the subapical hair cortex, without involvement of known upstream regulatory genes.

The keratin-associated proteins (KRTAPs) form a family of cysteine-rich and glycine-tyrosine repeat-rich proteins. Unique to mammals, they are a critical determinant of hair shaft and cuticle rigidity (Rogers et al. 2006; Jones et al. 2010; Fraser and Parry 2018). In our study, we found down-regulation of *Krtap* genes in the hair cortex, but not in the cuticle of *Hq* mutant mice. The pattern of down-regulation of murine hair keratin and *Krtap* genes in *Hoxc13* null mice (Potter et al. 2011) differs from the one observed in *Hq* mutant mice in two aspects: first, in *Hq* mutant mice the expression of genes encoding structural proteins gradually decreases between P5 and P12, while in *Hoxc13* null mice expression of structural genes is almost completely lost already on P5. Second, while the reduction of *Krt82* was 40-fold in *Hoxc13* null mice (Potter et al. 2011), the expression was preserved in *Hq* mutant mice. Thus, while *Foxn1* and *Hoxc13* mutations target hair cortex and cuticle alike, the *Hq* mutant allele causes alopecia by affecting specifically the hair cortex.

In humans, *AIFM1* mutations have been identified in patients suffering from severe X-linked mitochondrial encephalomyopathy (Ghezzi et al. 2010) and Cowchock syndrome (Rinaldi et al. 2012), both conditions affecting the nervous system and skeletal muscle. A mouse model of the R201 deletion identified in humans exhibit early defects primarily in the skeletal muscle (Wischhof et al. 2018). In this mouse model, we showed that aberrant AIF expression had no effect on the hair follicle, while the gradual loss of ETC in the *Tfam*^*EKO*^ mutant mouse does affect hair follicles (Bodemer et al. 1999). Similarly, the assembly and function of human mitochondrial complex III depends on the BCS1L protein, and mutations of BCS1L can cause Björnstad syndrome (Bénit et al. 2009), which is a combination of sensorineural hearing loss and pili torti, a condition in which the hair shafts are thin and broad, and twisted along their long axis (Rogers 1995). There is also other evidence that dysfunctional mitochondrial complex I may contribute to the *Aifm1*^*Hq/Y*^ pelage phenotype (Vahsen et al. 2004; Bénit et al. 2008; Kruse et al. 2008), since the alopecia in *Aifm1*^*Hq/Y*^ mice resembles the alopecia in mice with functional inactivation of the complex I subunit NDUFS4 (Kruse et al. 2008). Moreover, hair follicles engage in aerobic glycolysis, at least in vitro (Philpott and Kealey 1991; Kealey et al. 1994) and also require a controlled level of reactive oxygen species (ROS) generated by mitochondria for their normal development in vivo (Hamanaka et al. 2013). Thus, further studies will have to show by which mechanism the reduction of AIF protein contributes to the pelage defect in *Hq* mutant mice in vivo.

The pelage of *Aifm1*^*Hq/X*^ heterozygous female mice exhibited a surprisingly mild defect, although theoretically half of all cells in each *Aifm1*^*Hq/X*^ HF should suffer the same genetic deficit as all cells in *Aifm1*^*Hq/Y*^ hemizygous male HFs. The deformation of each individual *Aifm1*^*Hq/X*^ hair shaft seems to follow an all-or-none rule being either phenotypically normal or completely deformed like the hair shafts of the *Hq* mutant males. During anagen, each layer of the growing IRS and the hair shaft is replenished by lineage-restricted precursor cells originating from the HF matrix that form small clones of differentiated cells (Legué and Nicolas 2005; Legué et al. 2010). Hence, each layer of the hair follicle in *Aifm1*^*Hq/X*^ heterozygous female mice is a mosaic of cell clusters with normal and impaired mitochondria.

We found that *Aifm1 (R200 del)* caused a roughly 55% decrease of AIF protein in skin, presumably due to decreased protein stability, which is a milder reduction compared to the AIF loss in other tissues (Wischhof et al. 2018). The decrease of AIF in the skin of *Aifm1*^*HqY*^ hemizygous males was roughly 80%, confirming previous reports (Klein et al. 2002; Wischhof et al. 2018). These results suggest that there may be a critical threshold between a 55 and 80% reduction of AIF in the hair follicle to result in an all-or-none phenotype with respect to hair fragility. Apart from reduced AIF levels, we reported that the *Aifm1*^*Hq*^ mutation is associated with an ecotropic proviral integration that expresses a full-length proviral genome and a spliced *env* transcript. Thus, we wanted to investigate the possibility that the expression of *Hq* mutation-associated retroviral elements might play a role in the development of the mutant hair phenotype. Proviral integrations are associated with other pelage phenotypes, such as dilute (Jenkins et al. 1981; Copeland et al. 1983a), lethal yellow (Copeland et al. 1983b), or the *Plcd3*^*mNab*^/*del9(Pas)1*-associated alopecia (Runkel et al. 2012), even though the expression of viral elements may not be a contributory factor in these mutants. Given our observation that *Hq* mutation-associated retroviral elements are strongly expressed in *Hq* skin, we established an in vitro system to examine the role of the *Aifm1*^*Hq*^-associated ERV for the dysregulation of hair follicle *Krt* and *Krtap* genes. Even though our cell line Kera2 showed a predominantly epidermal gene expression profile, the forced expression of *Hoxc13* caused an elevated expression of *Krt84* and *Krtap33* genes, which are both expressed in the hair cortex in vivo. After transfection of the genomic sequence of the *Hq*-associated ERV, which lead to detectable ERV *env* expression, some clones specifically down-regulated the expression of hair cortex-specific *Krt84* and *Krtap33*, but not of epidermal *Krt5* or *Aifm1* genes. These data suggest that the ecotropic ERV in the *Aifm1* genomic locus might influence the expression of hair-associated structural genes on its own without altering *Aifm1* expression.

Repeatedly during mammalian evolution, retroviruses have integrated into the genomes and functioned as mutagenic mobile genetic elements. Upon infection of mammalian cells, retroviruses reverse transcribe their RNA genomes and subsequently integrate as DNA proviruses into the host genome. When proviruses integrate into the genomes of germ cells, they become novel genomic elements of the host that will be transmitted to the following generations as endogenous retroviruses, reviewed in Maksakova et al. (2006); Meyer et al. (2017); and Gagnier et al. (2019). Endogenous retroviruses (ERV) in mice make up about 8 to 10% of the host genome. They may be active or become inactive due to the continuous acquisition of mutations or deletions after genomic recombination (Buzdin et al. 2017; Meyer et al. 2017) and their transposition may be the cause of approximately 10% of new mutations (Maksakova et al. 2006). In this report, we have characterized the *Aifm1*^*Hq*^-associated provirus as an ecotropic full-length retrovirus almost identical in sequence to EMV30 (Triviai et al. 2014). ERVs can alter the their host’s transcription profile by viral promoter-mediated lateral activation of adjacent host genes, by viral enhancer-mediated cis-activation of cryptic promoters (Morishita et al. 1988), or by providing splice signals and polyA signals (Kapitonov and Jurka 1999). While such mechanisms may explain the down-regulation of *Aifm1* expression in the *Hq* mutant, they cannot explain the dysregulation of hair cortex genes which are located on autosomes at a remote genomic distance from the X-chromosomal *Aifm1* locus. Alternatively, ERVs may influence host gene expression by viral non-coding RNAs (Zhang et al. 2018) or by providing novel binding sites for transcription factors (Tsuruyama et al. 2011). ERV-derived proteins or protein fragments can also cause specific biological effects in the human placenta, the immune system, and the pathogenesis of neurodegenerative disease in mice (Apte and Sanders 2010; Lokossou et al. 2014). Our data obtained from Kera2 cells transfected with the full-length *Hq*-ERV provide evidence that expression of endogenous retroviral elements can interfere with hair keratins in vitro, which may be a contributory factor to the hair phenotype elicited in the mutant. Using this tool, future experiments with mutated and/or truncated forms of the viral genome will allow us to gain a more detailed understanding of the underlying molecular processes.

## Materials and methods

### Ethics statement

Animals were sacrificed according to §14.3 of the German law for the protection of animals (Tierschutzgesetz), with Thomas Franz and Daniele Bano holding the permission to sacrifice mice (file number 50.203.2-BN 6/02, 84-02.04.2014.A521, 84-02.04.2015.A007). Mice were killed by cervical dislocation avoiding unnecessary pain. A permission to hold and breed mice based on §11(1) of the relevant animal welfare law was granted on 21st of March, 2014 to Thomas Franz. All procedures were followed as requested by the competent authorities.

### Mouse work

B6CBACa *A*^*w*-*J*^/*A*-*Aifm1*^*Hq*^/J mice were purchased from The Jackson Laboratory (Bar harbor, Maine, USA) (JAX stock number: 000501). Mice were crossed once with C57BL/6 J mice and the offspring maintained by minimal inbreeding as one agouti and one non-agouti mouse line. Animals were kept in a 12-h light/dark cycle with food and water ad libitum. Mouse genotypes were determined by PCR on genomic DNA obtained from tail or ear biopsies. Mutant *Aifm1 (R200 del)* KI mice have been previously described (Wischhof et al. 2018). Throughout all experiments, *Aifm1*^*Hq/Y*^ samples were compared to samples from age-matched *Aifm1*^*X/Y*^ (wt), *Aifm1*^*Hq/X*^, or *Aifm1*^*(R200 del)/Hq*^ littermates, as indicated in the figure legends.

Animals were sacrificed by cervical dislocation and rupture of cervical blood vessels, before tissues were removed for fixation.

### Histology, immunohistochemistry, and in situ hybridization

Histology and immunofluorescence (IF) of paraffin-embedded tissue fixed in Bouin’s solution were performed as previously described (Runkel et al. 2008). For IF, heat-mediated antigen retrieval was performed using 1 mM EDTA (pH 9.0). Monoclonal rabbit antibody D39D2 against AIF (Cell Signaling Technology) was used at a dilution of 1:100, followed by Cy3-conjugated goat anti rabbit IgG F(ab)2 (Dianova, 111-165-006) at a dilution of 1:500.

Gene-specific cRNA probes used in Northern blot analyses and in situ hybridizations were generated by RT-PCR as described (Runkel et al. 2008). In situ hybridizations were performed as described (Runkel et al. 2004). Oligonucleotides used in this work are listed in Table 1.

**Table 1:** List of primers used for RT-PCR and cRNA probe synthesis

Gene	Product length spliced/genomic	For primer	Rev primer	Comment/Gene function	Rev primer + T7 (where applicable)
FoxN1	800	CTGGGCTCACCTCACTATCC	AGGTCAGTCCCAAGGTCTCC	Regulator	GGATCCTAATACGACTCACAGGTCAGTCCCAAGGTCTCC
Hoxc13	540	GGTGACGACCTGTCCTCCAG	TTCGGGTGGATTCCGTTATG	Regulator	
Msx2	350	GCAAGGATGGTGGACTTGCT	TCACCTCTGTACCGTGTGAGGA	Regulator	*Only for RT*-*PCR on Kera2 cDNA*
Msx2	530	GGCGGTGACTTGTTTTCGTC	CCTTAGCCCTTCGGTTCTGG	Regulator	GGATCCTAATACGACTCACCCTTAGCCCTTCGGTTCTGG
ß-Catenin	150	TACGAGCACATCAGGACACC	CTGCACAAACAATGGAATGG	Regulator	
Krt5	550	TGAGTGGGGAAGGAGTTGGA	GATGGGGTTCTGCTTTGGTG	Epidermal Krt	GGATCCTAATACGACTCACGATGGGGTTCTGCTTTGGTG
Krt10	260/900	TGGCGGTAGCTATGGAGGAG	CAGCACGTTGGCATTGTCAG	Epidermal Krt	
Krt16	240	CTGGATGGCGAGAATATCCA	ACCACCATGAGAGGGTAGGG	Epidermal Krt	GGATCCTAATACGACTCACACCACCATGAGAGGGTAGGG
Krt31	240	CCAGCAAGGCAAGAGATAGC	TTGTACGCTAGAACCTGGGG	Hair Krt	
Krt32	400	TGTAACCCTTGCTCCACTCC	AGTTTAGCCGAAAACACATGC	Hair Krt	
Krt33	380	CTGCTGGAGAGCGAGGACTG	GCAAGAAAGGGCAGAAGAAGC	Hair Krt	GGATCCTAATACGACTCACGCAAGAAAGGGCAGAAGAAGC
Krt34	300	TTTAGTTCAAATGCAGGAACG	TCGGGAGGTGGATGAAGC	Hair Krt	
Krt35	470	GGAGAAGGCATCCTCACTGG	GGAGCAGCATCCACCTCAAC	Hair Krt	
Krt36	260	TCAAGTCAGCACCCAGATCC	GGAATATTTCCAGGGGAACC	Hair Krt	
Krt71	390	TCCAAGTGTTCTGAGCCTGGGTAGC	AAAGAATACAAGAGAAGCCAAGG	Hair Krt	GGATCCTAATACGACTCACAAAGAATACAAGAGAAGCCAAGG
Krt72	400	ACATGAGGGAAGTGGTGGAG	TACCCGATAGTTCCCTGTGC	Hair Krt	
Krt73	600	ATCGCAGTCTCTGGATGAGC	CTTCTTGGAGCAGCAGAACC	Hair Krt	
Krt75	800	TACAGGAAGCTGCTGGAAGG	ATCAGCATTGCACACAGAGG	Hair Krt	
Krt82	400	GGGCACCGGTGAACTCTACA	CCCTCCTGGGAGAGAAGTGG	Hair Krt	GGATCCTAATACGACTCACCCCTCCTGGGAGAGAAGTGG
Krt84	420/1600	CTCACCCCGGATAGCAGTTG	TCCGGGCACATTTCTGTTCT	Hair Krt	GGATCCTAATACGACTCACTCCGGGCACATTTCTGTTCT
Krt86	400	CTGGTACTGCCCCTGCTGTC	GCTCTGGGTCACGTGGTTTC	Hair Krt	GGATCCTAATACGACTCACGCTCTGGGTCACGTGGTTTC
Krtap3-2	480	AGGTTTTGCTTATGTGATCCGGA	CAGGGGTTAAATTCTGCTTGCCTT	Hair Krtap	
Krtap3-3	300	CTTGCTTGGCACCAGTCTTC	AAACCCCAAACACCCAAAGA	Hair Krtap	GGATCCTAATACGACTCACAAACCCCAAACACCCAAAGA
Krtap4-7	460	TGAGGAGGGCTGTAGCCAAG	GAAGCTGGATCAAGGCATGG	Hair Krtap	GGATCCTAATACGACTCACGAAGCTGGATCAAGGCATGG
Krtap5-2	400	GTGGCTGTTCAGGAGGCTGT	CAGCTGGATTGGCAACAACA	Hair Krtap	GGATCCTAATACGACTCACCAGCTGGATTGGCAACAACA
Krtap6-1	360	AACCTCAACAACCAGCACC	TGCTGGTCATAAATGGATGC	Hair Krtap	
Krtap8-2	130	ACGGCAGCTACTACGGAGGT	GAATCGGGAGAATCCATATCC	Hair Krtap	GGATCCTAATACGACTCACGAATCGGGAGAATCCATATCC
Aifm1 5′	390	GAGGAGTGATCGCCGAAATG	AGTCCCTCCACCAATCAGCA	Aifm1 5′ probe	GGATCCTAATACGACTCACAGTCCCTCCACCAATCAGCA
Aifm1 3′	380	GGGGATGCTGCATGCTTCTA	TCCCCACCACAACTTTGTCC	Aifm1 3′ probe	GGATCCTAATACGACTCACTCCCCACCACAACTTTGTCC
Aifsh	470	TGTTTCTTTGGTGGGGATGG	ACCGCTGACTCCAACTGATT	Aifsh variant	
Hq-ERV	509	GGTAGACGGCATCTCTGCAT	TTGTGGGCATGTGTCACTTT	Hq-ERV probe	GGATCCTAATACGACTCACTTGTGGGCATGTGTCACTTT
ERV*gag*	1582	GGCTGACTGAGGCCAGAAAA	CCATACAGCCCTGGCTTGAC	Hq-ERV *gag*	
ERV*env*	1030	GTTCAGGAAGCAGCGACTCC	CAAAGCTCGACCAGGACACA	Hq-ERV *env*	

Transmitted light microscopy images were captured on a Leica DMRB microscope. Confocal fluorescence images were captured on a Nikon A1R confocal microscope using a 20× objective.

Ultrastructural investigations using transmission electron microscopy (TEM) were performed as previously described (Runkel et al. 2008), using a Leo910 TEM.

### Western blot analyses

Western blot analyses were carried out as described (Runkel et al. 2008). Goat antibody against AIF (Santa Cruz, sc-9416) was used at a dilution of 1:300. Goat antibody against actin (Santa Cruz, sc-1615) was used at a dilution of 1:30,000. Mouse monoclonal antibody against GAPDH (Hytest, 5G4MAb6C5) was used at a dilution of 1:500. POD-conjugated rabbit anti-goat IgG (Dianova, 305-036-003) or goat anti-mouse F(ab)2 (Dianova, 115-036-062) were used at 1:30,000 or 1:25,000 dilutions, respectively.

### Northern blot analyses

RNA blot hybridizations were performed and gene-specific cRNA probes were generated as previously described (Runkel et al. 2008). The list of gene-specific primers used to generate DIG-labeled cRNA probes is reported in Table 1. *Hq*-ERV-specific cRNA probes were generated from PCR products with 100% sequence identity to our cloned *Hq*-ERV genomic sequence.

### qRT-PCR analysis

Dorsal mouse skin biopsies were snap-frozen in liquid nitrogen and ground in a mortar for total RNA extraction using the NucleoSpin RNA midi kit (Macherey & Nagel) according to the manufacturer’s recommended protocol. RNA from Kera2 cells was isolated from a cell pellet collected by trypsin dissociation and centrifugation. mRNA was purified from 1 µg total RNA using the Oligotex mRNA mini kit (Qiagen) following the manufacturer’s recommended protocol. cDNA was synthesized from the resulting mRNA. Approximately 1 µl of skin cDNA or 2 µl of Kera2 cDNA were used for quantitative RT-PCR on a LightCycler™ 96 using the universal probe library protocol (both Roche), with primers and probes as listed in the table. Experiments were performed as biological triplicates and technical duplicates, expression levels were normalized to the respective wt samples, except for the *Hq*-Env expression, which was normalized to *Hq*-ERV-containing samples (*Hq*/Y skin or *Hq*-ERV-transfected Kera2 cells, as applicable). Gene-specific primers and probes are listed in Table 2.

**Table 2:** List of qRT-PCR primers and probes (Roche universal probe library)

Gene	Gene ID	For primer	Rev primer	UPL probe
Krt5-001	ENSMUST00000023709.5	CAGGACCTGGTGGAGGACTA	TCCAGCTCCACCTTGTTCAT	#89
Krt84-201	ENSMUSG00000044294.7	CACCTGCAAGACATCCTTGA	AAACTCATTCTCGGCATTGG	#10
Krtap3-3-001	ENSMUSG00000069722.4	TGAAGAGATCAAAGCCAACCA	CAGCGGCAGGACTTGTCT	#67
Aifm1-001	ENSMUSG00000036932.14	TCCAAGCACGTTCTAACATCTG	GCCTTCGACCCAACTTTATATC	#89
Hq-Env	n.a.	GGTTCACGAGCTTTGGAAAA	GAGACGTATAGGCGCAATCC	#10
Gapdh-001	ENSMUST00000118875.7	GGGTTCCTATAAATACGGACTGC	CCATTTTGTCTACGGGACGA	#52

### Molecular cloning

To generate a viral vector for cell immortalization, we sequentially cloned a blasticidin resistance gene (BlaR) followed by a modified T2A linker (PCR amplification from pDEST51 using primers FR2572 and FR2574) and a truncated temperature-labile SV40 tsA58T (PCR from pZipneotsA58 using FR2575 and FR2571; pZipneotsA58 was a kind gift from P. Jat. (Jat and Sharp 1989)) into the p50-M-X-neo retroviral plasmid (Laker et al. 1998). In the resulting vector p50MBla-tsA58T, the viral MeSV LTR drives the expression of BlaR-T2A-SV40tsA58T (Primers for cloning are listed in Table 3).

**Table 3:** List of genotyping and cloning primers

Primer ID	Primer sequence	Description
FR470	AGTGTCCAGTCAAAGTACCGGG	Aifm1 intron 1–2 For (Klein et al. 2002)
FR471	CTATGCCCTTCTCCATGTAGTT	Aifm1 intron 1–2 Rev (Klein et al. 2002)
FR472	CCAGAAACTGTCTCAAGGTTCC	Hq-ERV U3 LTR For (Klein et al. 2002)
FR2571	TTGGATCCTTATGTTTCAGGTTCAGGGGGAGG	SV40tsA58 Rev with BamHI site
FR2572	AAAAGCGGCCGCATGGCCAAGCCTTTGTCTCAAGA	BlaR-For with NotI site
FR2574	TTGAATTCGACGTCACCGCAACTAGTCAGACTGCCCTCCCACA CATAACCAG	BlaR-Rev + partial T2A with EcoRI site
FR2575	TCGAATTCAATCCTGGCCCAATGCCATCTAGTGATGATGAGGC TACTGC	SV40tsA58 For + partial T2A with EcoRI site
FR3134	GGTTCCTATTTCTAGGACCTTTAGG	Aifm1 intron 5–6 For (Wischhof et al. 2018)
FR3135	CCATTGCTCCCAAGAAAGG	Aifm1 intron 5–6 For (Wischhof et al. 2018)

The murine HoxC13 expression vector expressing a neomycin resistance was purchased from Origene, BioCat (MR226373-OR).

To clone the *Hq* allele-linked ERV genome, a 9.3-kbp-long DNA fragment was amplified from genomic DNA of *Aifm1*^*Hq/Y*^ mice using primers FR470 and FR471 derived from the first intron of the *Aifm1* gene as described in Klein et al. (2002). The amplified PCR fragment was then ligated into the TOPO XL2 cloning vector (ThermoFisher). The resulting plasmid pTOPO-Hq-ERV (Addgene #164088) was amplified in Stbl3 bacteria and fully sequenced (GenBank MW030280).

### Tissue culture

GP + E-86 ecotropic helper cells (Markowitz et al. 1990) were transfected with p50MBla-tsA58T and clones selected in 1 µg/ml blasticidin. Blasticidin-resistant clones were growth arrested by treatment with mitomycin C for 4 h. Primary keratinocytes were isolated from the skin of 2-day-old C57BL/6 J mice and co-cultured with growth-arrested p50MBla-tsA58T-transfected producer cells for immortalization. The keratinocytic cell line Kera2 showed sustained growth at the permissive temperature of 32 °C in CaCl_2_-free DMEM. In the same way, a keratinocytic cell line Hq-Kera was established from *Hq* mutant males.

To generate *Hq*-ERV transfected Kera2 clones, the entire insert was excised from pTOPO-Hq-ERV using *Pme*I and *Not*I. The excised insert was gel-purified and co-transfected into mammalian Kera2 cells using Xfect transfection reagent (Takara) together with vector pPur (Clontec). After transfection a single-cell suspension was prepared using trypsin and the cells were plated at limiting dilutions in 96-well plates. The transfected cells were selected in 1 µg/ml puromycin and clones examined by genomic PCR for the presence of the *Hq*-ERV.

## Supplementary Information

Below is the link to the Supplementary Information.

Supplementary material 1 (DOCX 173 kb)

## Data Availability

Materials used in this study will be sent to interested researchers upon request.
