# Supplementary material for: Alopecia in Harlequin mutant mice is associated with reduced AIF protein levels and expression of retroviral elements
Source: Mamm Genome. 2020 Dec 26;32(1):12–29. doi: 10.1007/s00335-020-09854-0 (PMC7878237; doi:10.1007/s00335-020-09854-0)
Supplement: Supplementary file 1 — Supplementary material 1 (DOCX 173 kb) [file 335_2020_9854_MOESM1_ESM.docx]

#
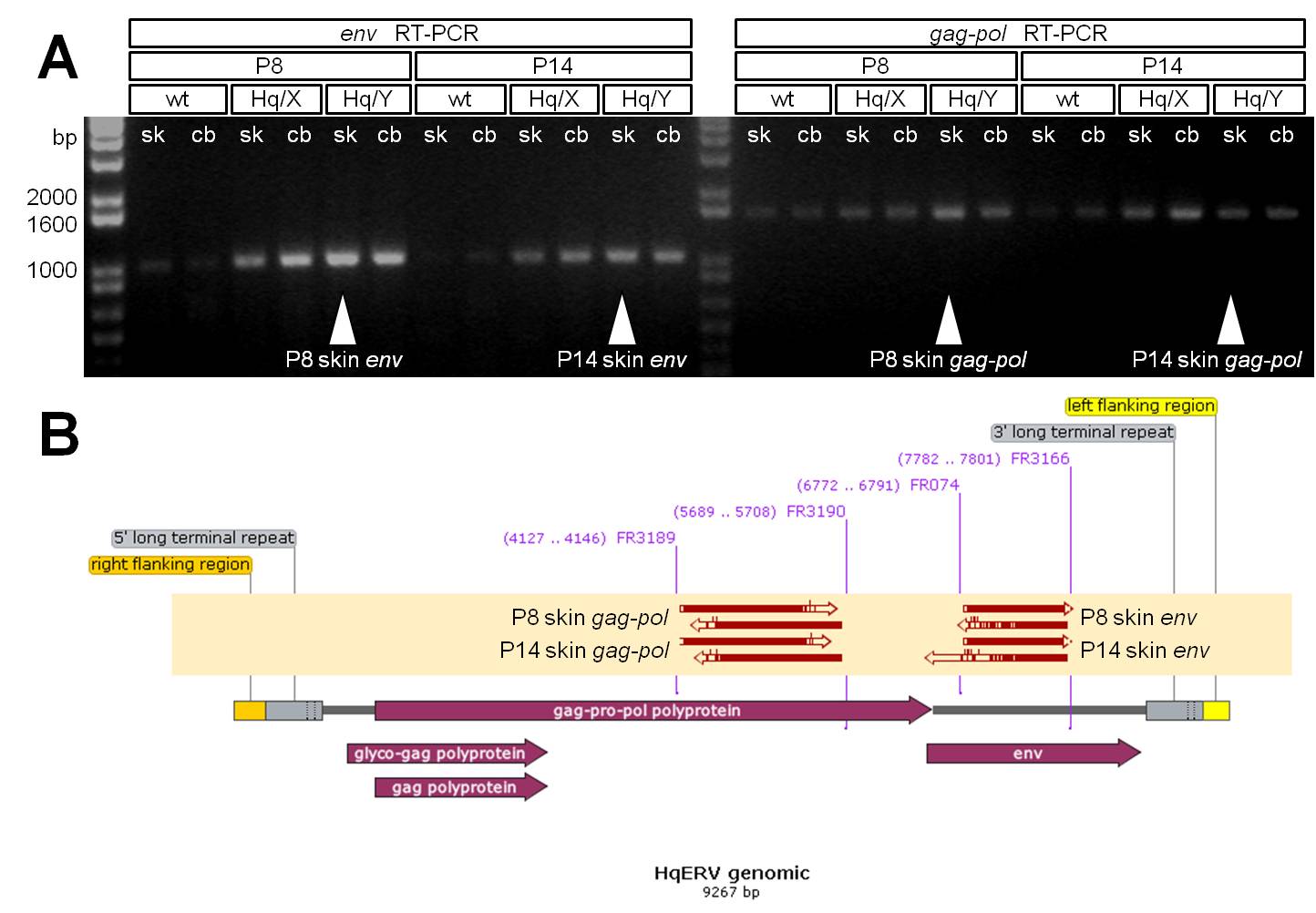


**Supplementary Figure S1**: A, Retroviral transcripts can be readily amplified from *Aifm1^Hq/Y^* and *Aifm1^Hq/X^* mutant skin (sk) and cerebellum (cb) cDNA samples, but are hardly detectable in wt littermate control cDNAs. B, RT-PCR products indicated in A were aligned to the *Hq*-ERV genomic sequence (cf. Fig. 6A). 100% sequence identity demonstrates that the transcripts are derived from the *Aifm1^Hq^* allele-associated retroviral insertion. Primers indicated in the figure were used for amplification and sequencing (FR074, GTTCAGGAAGCAGCGACTCC; FR3166, CAAAGCTCGACCAGGACACA; FR3189, GGCTGACTGAGGCCAGAAAA; FR3190, CCATACAGCCCTGGCTTGAC). Sequences were aligned and map was generated using SnapGene software.
